# Supplementary material for: Ultrasound-controllable engineered bacteria for cancer immunotherapy
Source: Nat Commun. 2022 Mar 24;13:1585. doi: 10.1038/s41467-022-29065-2 (PMC8948203; doi:10.1038/s41467-022-29065-2)
Supplement: Supplementary file 1 — Supplementary Information [file 41467_2022_29065_MOESM1_ESM.pdf]

# Supplementary Information

Ultrasound-controllable engineered bacteria for cancer immunotherapy

Mohamad H. Abedi[1,#,&], Michael S. Yao[1,#], David R. Mittelstein[2], Avinoam Bar-Zion[3], Margaret B. Swift[3], Audrey Lee-Gosselin[3], Pierina Barturen-Larrea[3], Marjorie T. Buss[3], Mikhail G. Shapiro[3,4,*]

**Affiliations:**
[1] Division of Biology and Biological Engineering
[2] Division of Engineering and Applied Sciences
[3] Division of Chemistry and Chemical Engineering
[4]Howard Hughes Medical Institute
California Institute of Technology
Pasadena, CA, USA 91125

[#]These authors contributed equally
*Correspondence should be addressed to MGS:
Email: mikhail@caltech.edu
Phone: 626-395-8588
1200 E. California Blvd, MC 210-41, Pasadena, CA 91125
[&]Present address: Department of Biochemistry, Institute for Protein Design and Howard Hughes Medical Institute, University of Washington, Seattle, WA, USA 98195

**Table of Contents**
Supplementary Figures 1-9

**FIGURES**

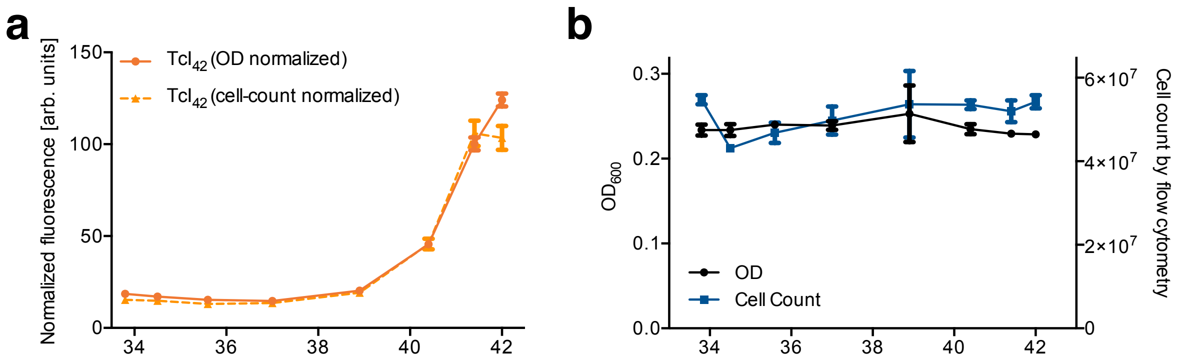


**Supplementary Figure 1 | Evaluation of different methods to normalize fluorescence from activated cells.** (**a**) EcN cells carrying the $TcI_{42}$ plasmid evaluated in **Fig. 1b** were analysed again, and their signal was normalized either with OD measurements or flow cytometry cell counts. (**b**) Raw measurement of $OD_{600}$ and cell count by flow cytometry. These results indicate that the fold changes observed is circuit activation are not a consequence of different OD measurements at different temperatures. Furthermore, normalizing by flow cytometry counts provided comparable results to OD normalization, validating that OD serves as a reasonable surrogate for total cells count. n=4 biologically independent replicates. All source data are provided as a Source Data file. Error bars represent (±SEM). Cytometry gating strategy is shown in Supplementary Fig. 9.

**a**

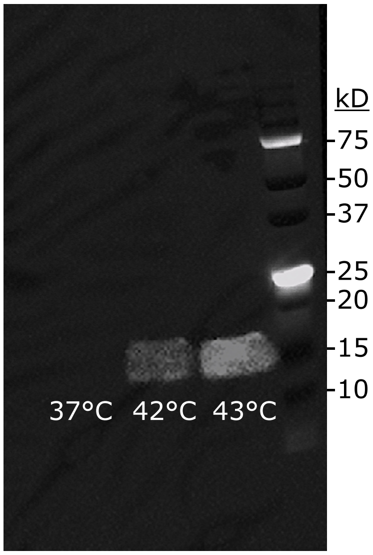


**Supplementary Figure 2 | Western blot to assay for the release of αCTLA-4 upon thermal activation.** (**a**) Unmodified image of the western blot shown in **Fig. 3c.** The image in Fig. 3c was cropped and inverted to make it fit better into the figure presentation. This experiment was repeated independently three times with similar results.

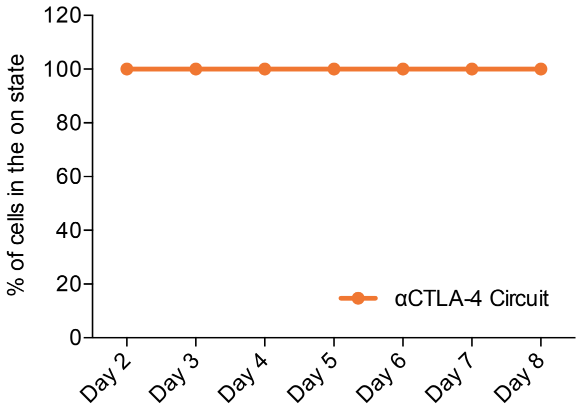


**Supplementary Figure 3 | Stability of gene expression in thermally induced circuits.** EcN cells were transformed with the αCTLA-4 therapeutic circuit from Figure 3 and thermally induced at 43°C for 1 hour with the five-minute pulsing scheme. The following day four colonies were picked and propagated to assess circuit stability. These cells were diluted every day by a factor of 1000x and simultaneously plated to assess the number of cells in the on state from each colony by counting GFP positive cells. We could not find any evidence of burden on the cells which is typically reflected in mutational escape. n=4 biological replicates. All data points were 100% GFP positive. n=4 biologically independent replicates. All source data are provided as a Source Data file.

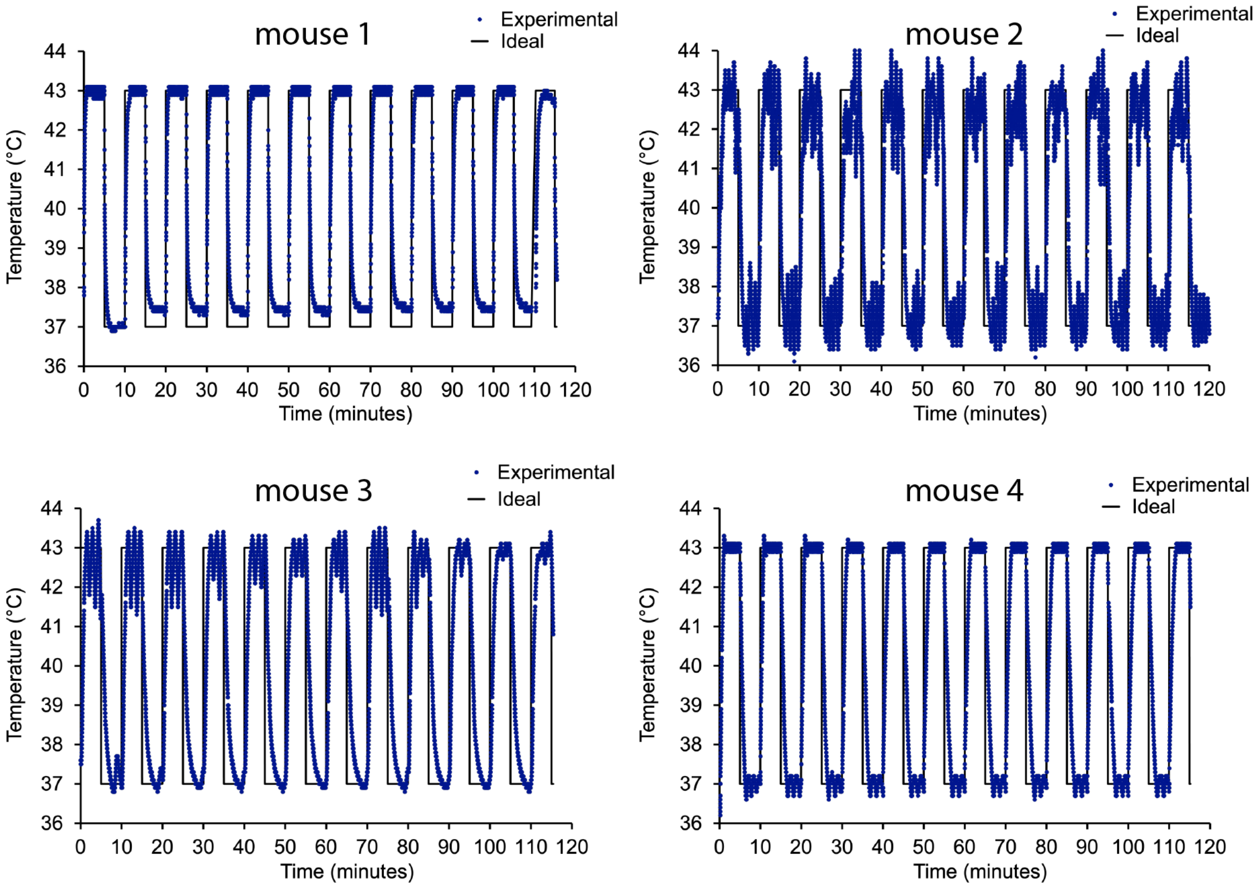


**Supplementary Figure 4 | FUS oscillatory heating of tumors.** Tumor temperature measurements in four individual mice treated with alternating 5-min steps between 37 °C and 43 °C under feedback control. All source data are provided as a Source Data file.

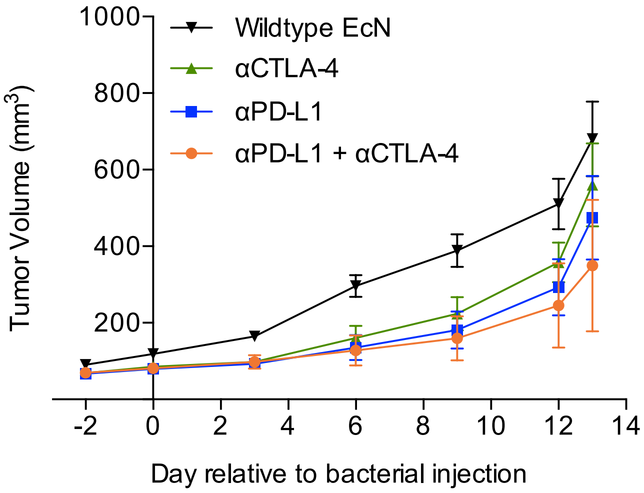


**Supplementary Figure 5 | Assessing the effect of combination checkpoint therapy.** EcN cells were transformed with the αCTLA-4 or αPD-L1 therapeutic circuit from Figure 3 and thermally induced at 43°C for 1 hour with the five-minute pulsing scheme. The following day activated colonies were picked and propagated before being injected into mice bearing A20 tumors. Mice were injected with αCTLA-4 EcN; αPD-L1 EcN; αCTLA-4 and αPD-L1 EcN at 1:1 ratio; or wildtype EcN. Tumor sizes were measured over two weeks. Four mice were used to acquire the data for the αPD-L1 + αCTLA-4 and αPD-L1 conditions. Five mice were used for the αCTLA-4 and Wildtype EcN groups. All source data are provided as a Source Data file. Error bars represent (±SEM).

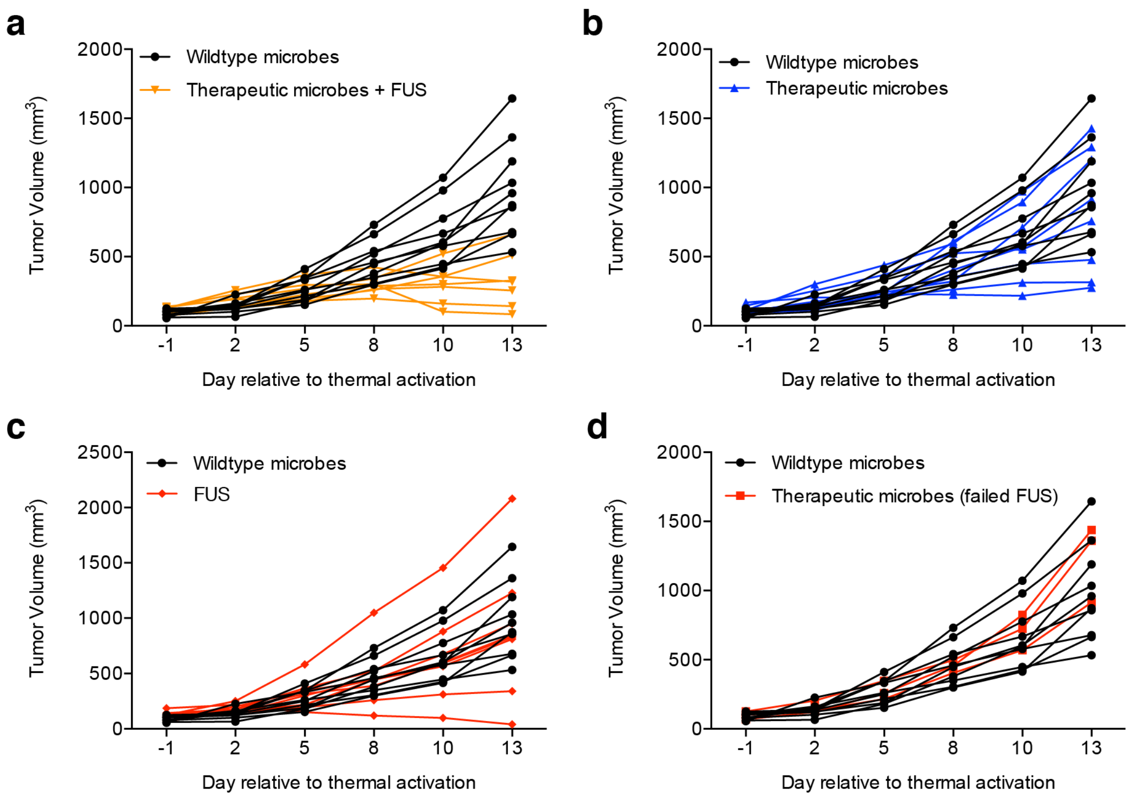


**Supplementary Figure 6 | Individual growth curves of tumors analysed in Fig. 4c.** Individual growth curves of all the tumors analysed and plotted in Fig. 4c. All conditions plotted relative to animals injected with wildtype microbes. All source data are provided as a Source Data file. [10,7,8,8,3] animals were used for the wildtype, Therapeutic +FUS, Therapeutic, FUS and Therapeutic (failed FUS) conditions respectively.

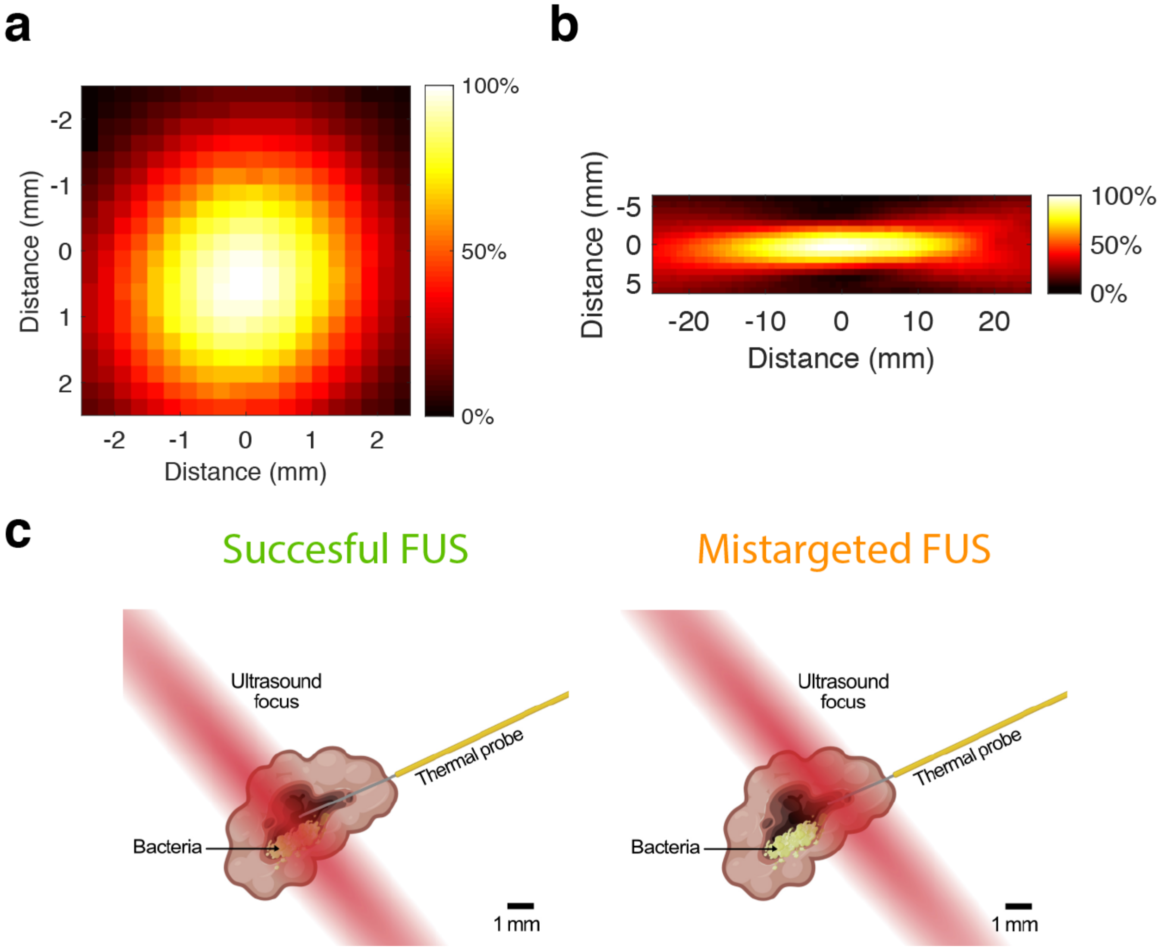


**Supplementary Figure 7 | Characterization of the FUS beam.** (**a**) Normalized peak-negative pressure field of the 670 kHz transducer used in this study, measured using a fiber-optic hydrophone in the transverse plane, orthogonal to direction of propagation. (**b**) Normalized peak-negative pressure measured in the longitudinal plane, along the direction of FUS propagation. (**c**) Illustration of the typical cross-sectional dimensions of the tumors treated in this study relative to the area covered by the FUS focal zone (3.5 mm lateral and 35 mm longitudinal full width at half maximum pressure). The illustration includes a hypothetical sub-tumoral distribution of bacterial cells, showing how the ultrasound heating setup used in this study could correctly (left) or incorrectly (right) target the cells for activation.

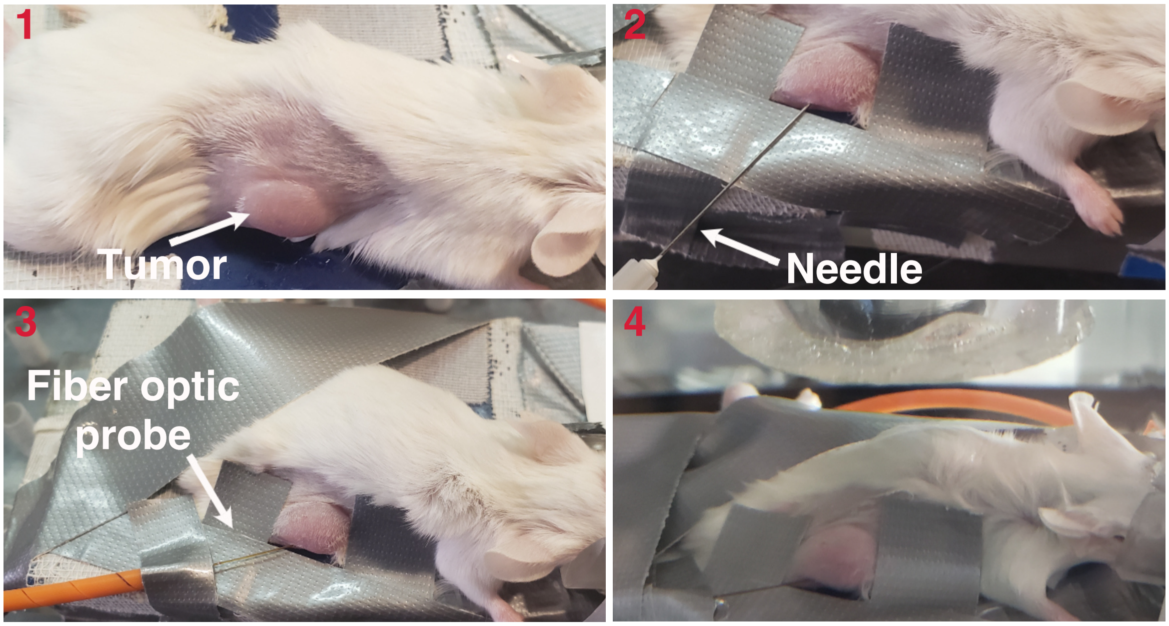


**Supplementary Figure 8 | Fiber-optic probe insertion procedure**. To insert the probe into tumors we followed the following procedure: (1) shave the tumor before the mouse is placed on a holder, (2) insert a 25-gauge needle into the tumor to guide the fragile thermal probe, and (3) insert the fiber-optic probe into the path created by the needle and secure the probe with duct tape.

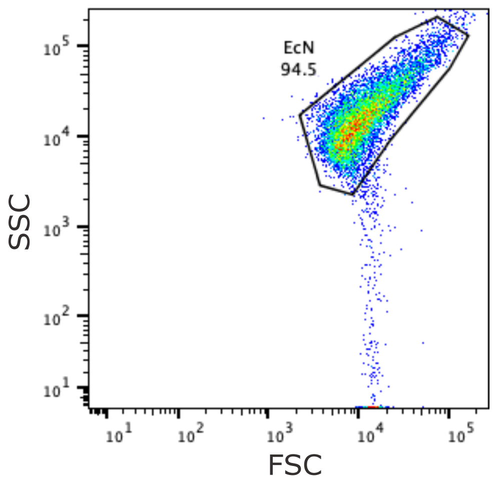


**Supplementary Figure 9 | Flow cytometry gating strategy for Supplementary Figure 1b.**
