# Supplementary material for: Ultrasound-controllable engineered bacteria for cancer immunotherapy
Source: Nat Commun. 2022 Mar 24;13:1585. doi: 10.1038/s41467-022-29065-2 (PMC8948203; doi:10.1038/s41467-022-29065-2)
Supplement: Supplementary file 2 — Reporting Summary [file 41467_2022_29065_MOESM2_ESM.pdf]

nature portfolio

Corresponding author(s): Mikhail G. Shapiro

Last updated by author(s): Jan 30, 2022

Reporting Summary

Nature Portfolio wishes to improve the reproducibility of the work that we publish. This form provides structure for consistency and transparency in reporting. For further information on Nature Portfolio policies, see our Editorial Policies and the Editorial Policy Checklist.

Statistics

For all statistical analyses, confirm that the following items are present in the figure legend, table legend, main text, or Methods section.

n/a	Confirmed	
[ ]	[x]	The exact sample size (n) for each experimental group/condition, given as a discrete number and unit of measurement
[ ]	[x]	A statement on whether measurements were taken from distinct samples or whether the same sample was measured repeatedly
[ ]	[x]	The statistical test(s) used AND whether they are one- or two-sided<br>*Only common tests should be described solely by name; describe more complex techniques in the Methods section.*
[x]	[ ]	A description of all covariates tested
[x]	[ ]	A description of any assumptions or corrections, such as tests of normality and adjustment for multiple comparisons
[ ]	[x]	A full description of the statistical parameters including central tendency (e.g. means) or other basic estimates (e.g. regression coefficient) AND variation (e.g. standard deviation) or associated estimates of uncertainty (e.g. confidence intervals)
[ ]	[x]	For null hypothesis testing, the test statistic (e.g. F, t, r) with confidence intervals, effect sizes, degrees of freedom and P value noted<br>*Give P values as exact values whenever suitable.*
[x]	[ ]	For Bayesian analysis, information on the choice of priors and Markov chain Monte Carlo settings
[x]	[ ]	For hierarchical and complex designs, identification of the appropriate level for tests and full reporting of outcomes
[x]	[ ]	Estimates of effect sizes (e.g. Cohen's d, Pearson's r), indicating how they were calculated

Our web collection on statistics for biologists contains articles on many of the points above.

Software and code

Policy information about availability of computer code

Data collection	Custom code used to operate the focused ultrasound system https://github.com/drmittelstein/thermal_control
Data analysis	GraphPad Software, Inc. V6

For manuscripts utilizing custom algorithms or software that are central to the research but not yet described in published literature, software must be made available to editors and reviewers. We strongly encourage code deposition in a community repository (e.g. GitHub). See the Nature Portfolio guidelines for submitting code & software for further information.

Data

Policy information about availability of data

All manuscripts must include a data availability statement. This statement should provide the following information, where applicable:
- Accession codes, unique identifiers, or web links for publicly available datasets
- A description of any restrictions on data availability
- For clinical datasets or third party data, please ensure that the statement adheres to our policy

Plasmids will be made available through Addgene upon publication.

Field-specific reporting

Life sciences study design

All studies must disclose on these points even when the disclosure is negative.

Sample size	This sample size was chosen based on preliminary experiments indicating that it would be sufficient to detect significant differences in mean
Data exclusions	Mice that did not have microbial cells in their tumors were excluded from the study. Three mice that did not successfully activate with HIFU were removed from our analysis of tumor growth.
Replication	All experiments were replicated on different days and starting with different biological samples at least twice.
Randomization	Mice were randomly allocated in this study
Blinding	Tumor measurements were performed while being blinded to group allocation. Blinding was not required for any other experiments because they did not allow for subjective judgment.

Reporting for specific materials, systems and methods

We require information from authors about some types of materials, experimental systems and methods used in many studies. Here, indicate whether each material, system or method listed is relevant to your study. If you are not sure if a list item applies to your research, read the appropriate section before selecting a response.

Materials & experimental systems

n/a	Involved in the study	
☐	☒	Antibodies
☐	☒	Eukaryotic cell lines
☒	☐	Palaeontology and archaeology
☐	☒	Animals and other organisms
☒	☐	Human research participants
☒	☐	Clinical data
☒	☐	Dual use research of concern

Methods

n/a	Involved in the study	
☒	☐	ChIP-seq
☐	☒	Flow cytometry
☒	☐	MRI-based neuroimaging

Antibodies

Antibodies used	mouse anti-His (IgG1 κ H3) antibody (Santa Cruz Biotech, sc-8036), mouse IgG kappa binding protein (m-IgGκ BP) conjugated to Horseradish Peroxidase (HRP) (Santa Cruz Biotech, sc-516102)
Validation	Antibodies validated by manufacturer and provided data on use in Western blots.

Eukaryotic cell lines

Policy information about cell lines

Cell line source(s)	A20 [A-20] (ATCC® TIB-208™) from the American Type Culture Collection
Authentication	Authentication was provided by ATCC. We do not know the method they used. We did not perform additional authentication assays ourselves.
Mycoplasma contamination	The cell line was not tested for mycoplasma contamination
Commonly misidentified lines (See ICLAC register)	No commonly misidentified lines were used

Animals and other organisms

Policy information about studies involving animals; ARRIVE guidelines recommended for reporting animal research

Laboratory animals	8-12 week-old BALB/c female mice were purchased from Jackson Laboratory. Information on housing provided in manuscript under animal procedures.
Wild animals	no wild animals were used in this study
Field-collected samples	no field collected samples were used in the study.
Ethics oversight	All animal procedures were performed under a protocol approved by the California Institute of Technology Institutional Animal Care

and Use Committee (IACUC).

Note that full information on the approval of the study protocol must also be provided in the manuscript.

Flow Cytometry

Plots

Confirm that:

- [ ] The axis labels state the marker and fluorochrome used (e.g. CD4-FITC).
- [ ] The axis scales are clearly visible. Include numbers along axes only for bottom left plot of group (a 'group' is an analysis of identical markers).
- [ ] All plots are contour plots with outliers or pseudocolor plots.
- [ ] A numerical value for number of cells or percentage (with statistics) is provided.

Methodology

Sample preparation	EcN cells were resuspended in cold PBS + 0.5% BSA (filtered with a 0.2 micron filter) to prevent clumping and were run at 3 different dilutions (targeting 1e6, 1e7, and 1e8 cells/mL)
Instrument	MACSQuant VYB
Software	FlowJo
Cell population abundance	Cells were abundant and the instrument was thoroughly cleaned to ensure that there are no counts being detected from debris.
Gating strategy	scattering

- [x] Tick this box to confirm that a figure exemplifying the gating strategy is provided in the Supplementary Information.
